# Innovation and Entrepreneurship Strategies of Teachers and Students in Financial Colleges and Universities Under the Direction of Food Security

**DOI:** 10.3389/fpsyg.2022.848554

**Published:** 2022-05-18

**Authors:** Guan Haojie

**Affiliations:** School of Economics and Trade, Henan University of Technology, Zhengzhou, China

**Keywords:** food security, innovation and entrepreneurship, TOPSIS analysis method, Internet of Things monitoring technology, entrepreneurship development

## Abstract

This study aims to better promote the innovation and entrepreneurship of teachers and students in finance and economics colleges and universities in terms of food security. Based on the relevant theories such as food security, innovation and entrepreneurship, the questionnaire was used to investigate the issues related to food security of teachers and students in colleges and universities. Next, the Technique for Order Preference by Similarity to an Ideal Solution (TOPSIS) analysis method was introduced to evaluate the safety metrics of the response subsystem. Finally, Internet of Things (IoT) monitoring technology was applied to grain growth and sales. The research results show that college teachers and students have a disjointed cognition of food security as important talents in innovation and entrepreneurship. After the TOPSIS analysis method is used to study, when college teachers and students choose to start a business in food security, they must pay attention to the changes in economic factors. The IoT monitoring technology is applied to grain growth and sales, and the monitoring technology of the IoT could reduce the mortality rate of corn seedlings to 3.59%. The mortality rate of seedlings is much higher than that of farmers relying on their own experience to grow corn. Therefore, this has great reference significance for the innovation and entrepreneurship development of college teachers and students in food security at this stage.

## Introduction

China has repeatedly emphasized that food security is a national plan, and that food production and population growth must be combined since the 19th National Congress of the Communist Party of China. This shows that food security is not only a matter of people’s livelihood, but also an important guarantee for economic operation (Scott et al., 2020; Ik et al., 2021). With the continuous improvement of urbanization, more and more young people choose to make a living in cities. This leads to the outflow of labor resources, low education level of the overall labor force in rural areas and backward farming technology (Kim and Choi, 2020). It is thus an important task to solve the current situation of food security to cultivate high-quality talents for grain planting.

Qian et al. (2018) stated that food security is the key to the development of ecological agriculture. They believed that the relationship between food security and ecological agriculture is the relationship between content and form (Qian et al., 2018). Chen (2019) proposed that grain is the basis of rural revitalization, and ensuring the normal production of grain is the basis of national governance (Chen, 2019). Cater (2020) proposed that children’s absorption of nutrients can indirectly reflect the country’s level of food security. Coates (2020) proposed that the internal logic of food security should be analyzed from the area, output, and *per capita* amount of food production (Coates, 2020). Hu (2020) selected 10 indicators from the level of agricultural science and technology, agricultural production, and climate conditions to evaluate food security (Hu, 2020). These experts and scholars put forward their own analysis strategies on food security and the importance of current food security from different angles. Therefore, how to implement food security in the innovation and entrepreneurship projects of college teachers and students is the common concern of all.

For the cultivation of talents in colleges and universities, attention must be paid to fundamentally improving the management level of food security. Their choice of entrepreneurship and innovation in food security needs to be further guided. According to the needs of the actual development level of agriculture and economy, questionnaires are conducted between teachers and students in colleges and universities. Next, the Technique for Order Preference by Similarity to an Ideal Solution (TOPSIS) analysis method is introduced to analyze the core of food security issues. Finally, Internet of Things (IoT) monitoring technology is of great help to the development of food security. This provides high-quality methods and strategies for college teachers and students in food security innovation and entrepreneurship. The innovation lies in the combination of theory and reality, and the latest policy of the development of the times. Through the ideological guidance of “Mass Entrepreneurship and Innovation,” it has continuously injected fresh vitality into China’s food safety production. The TOPSIS analysis method was used to study and analyze the current state of grain production. Finally, the corresponding conclusions were drawn.

## Materials and Methods

### Theories Related to Innovation and Entrepreneurship by Food Security

Food is the material basis for human survival (Polychronou et al., 2020). Food security refers to the fact that everyone can buy and afford the food they need without being affected by time and space (Luu et al., 2020). The food must not cause any harm to the human body first, and secondly it can satisfy the nutritional needs (Vikram et al., 2020).

Grain self-sufficiency rate refers to the indicator that the food supply in the region meets local demand. The quotient of the total amount of grain in the area in that year and the total grain demand of the local people in that year is the local grain self-sufficiency rate (D'Agata et al., 2021; Wieser et al., 2021). The food self-sufficiency rate is an important indicator to measure the food security of the place. If the food self-sufficiency rate of a place is less than 90%, it indicates that there is a problem with food security in that place. The self-sufficiency rate of food in a place is between 90 and 95%, indicating that the food security of the place is basically safe. The self-sufficiency rate of grain in a place is higher than 95%, indicating that the place can already rely on local grain to achieve self-satisfaction (Dawuda et al., 2020). Meanwhile, the main factors affecting grain production are concentrated in the following aspects.

#### Shortage of Labor

In modern society, the economic level is constantly improving, and the traditional production method of “men tilling the farm and women weaving” no longer exists. Nowadays, the young people are more willing to make a living in cities. The elderly and women basically farm at home. This poses great challenges to food production (Soylu et al., 2020).

#### Decreased Arable Lands

Since the 21st century, factories and high-speed railways have been developed in many places, and a large number of arable lands has been expropriated (Russo et al., 2021). In the face of natural and man-made calamities, no one has a better way to avoid it. This has severely affected the enthusiasm of farmers. Meanwhile, the soil has a “shelf life,” and planting crops on a piece of land will reduce the nutrients of the soil (Makhmudova, 2021).

#### Investment of Human Capital

At present, there is an increased consciousness of food security among teachers and students in colleges and universities, and they have been out of touch with the reality of food security (Loconsole and Santamaria, 2021). Theodore W. Schultz said that “for economic growth, the improvement of human resources is more important than the increase of material capital” (Kartikaningsih et al., 2021). As high-quality talents, teachers and students in colleges and universities should be guided to invest in the development of food safety production (Leonardo, 2021).

#### Advances in Science and Technology

At this stage, in terms of food planting, a certain foundation has been obtained both in terms of technology and talents. “Technology is the primary productive force,” and it is everyone’s common expectation to use some advanced technologies to improve farming efficiency.

**Figure 1:**
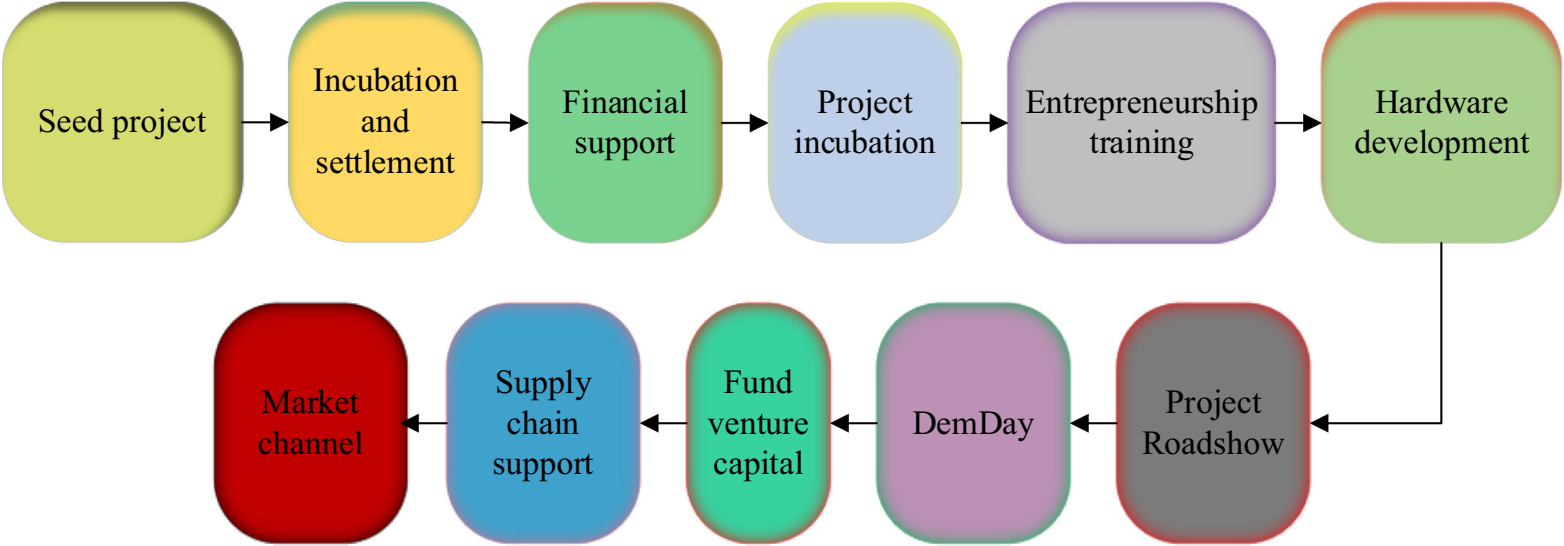
Innovation and entrepreneurship process of food safety.

**Figure 2:**
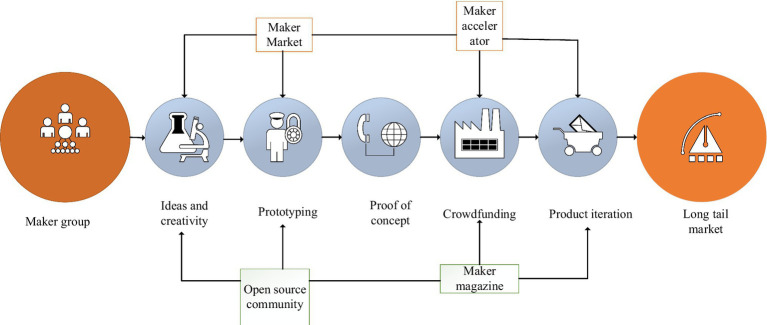
The operation mode of innovation and entrepreneurship.

Innovation and entrepreneurship refer to entrepreneurial activities that use a certain point or several innovations in technology, brand, service, management, etc. In 2014, Premier Li Keqiang put forward the initiative of “mass entrepreneurship and innovation” at the World Economic Forum meeting, after which there was a wave of entrepreneurship throughout the country. At present, the typical entrepreneurial operation model and operation process of food safety in China are shown in Figures 1, 2.

### Proposed Research Methods in Food Security Issues

#### Documentary Data Method

Firstly, CNKI and Google Scholar were used to query data. Secondly, many articles and works from columnists and related Internet information were cited (Ducos et al., 2021). In addition, platforms such as China Food Net and the Bureau of Statistics were used to find relevant information. Finally, some journals and books related to this article in the school library were also used, such as *Ecological Farm Documentary*, and *The Road to Agricultural Modernization with Chinese Characteristics*. The collection and summary of this series of data provide a favorable theoretical basis for the research ideas and methods of the proposed method (Moghaddam et al., 2020; Tomaszewska et al., 2021).

#### Expert Interview Method

By visiting food security experts, conducting invitation interviews with experts in related fields, and asking these experts for their views on the development of food security in recent years. Listening to experts’ suggestions and opinions on the development of food security will increase the scientific and rationality of the proposed method (Panseri et al., 2020).

#### Comparative Analysis Method

It refers to the multi-party comparison of two or more research objects, with the purpose of identifying the similarities and differences between the research objects and continuously increasing food production (Bomba and Susol, 2020; Long, 2020).

#### Questionnaire Method

**Figure 3:**
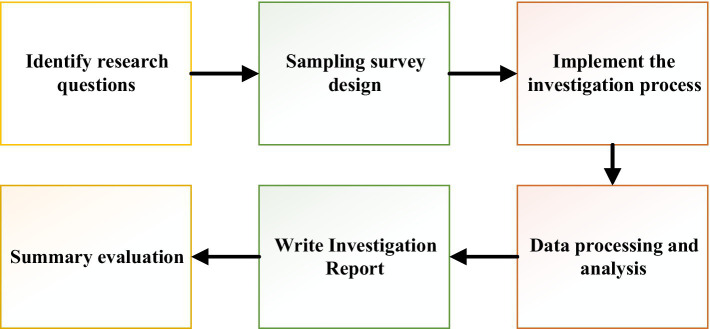
Questionnaire survey steps.

In this study, the questionnaire were used to set corresponding questions to understand the development status of food security. A total of 310 questionnaires were randomly distributed to scholars on food security twice in June 2019 and August 2020. In order to ensure the scientific nature of the questionnaires, the questionnaires were discussed with experts in related fields before the distribution, and the unreasonable points in the questionnaires are revised. The face-to-face distribution and on-site recovery were adopted to ensure the corresponding recovery rate. A total of 270 questionnaires were recovered and 248 valid questions were retained, with a recovery rate of about 87.1% and the effective recovery rate of 91.85%. The specific investigation steps are shown in Figure 3.

In Figure 3, the adopted questionnaire survey method is roughly divided into six steps. Firstly, the main research question is identified. The samples are then subjected to a sample survey design. Secondly, after the survey is completed, the data is collected, aggregated, processed, and analyzed. Finally, a research report is written and the results are summarized and output according to the data results.

In order to make the results of the questionnaire more accurate, the validity test of the systematic error variance is introduced, as shown in Eq. (1).


(1) $$r=\frac{\sum XY-\frac{\sum X\sum Y}{N}}{\sqrt{\sum X^{2}-\frac{{\left(\sum X\right)}^{2}}{N}\sqrt{\sum Y^{2}-\frac{{\left(\sum Y\right)}^{2}}{N}}}}$$

In Eq. (1), *r* represents the reliability coefficient. *X* represents a dependent variable. *Y* represents independent variable. *N* represents the number.

According to Eq. (1), the score difference of actual numbers is obtained, as shown in Eq. (2).


(2) $$\alpha =\frac{K}{K-1}\left(1-\frac{\sum_{i=1}^{K}\sigma_{Yi}^{2}}{\sigma_{X}^{2}}\right)$$

In Eq. (2), *α* represents a coefficient. *K* represents the quantity. The meaning of other letters is the same as Eq. (1).

### TOPSIS Analysis Method

The TOPSIS analysis method is now an important index used to evaluate the pros and cons of selected objects (Kolanowski et al., 2021). Then, the result of the selected object was compared with the optimal solution and the worst solution in the target (Drgoi and Rdulescu, 2021). The closer the result is to the optimal solution and the farther the worst solution is, it means that the object is the best; otherwise, it is sub-optimal. Finally, the results are ranked, the first is the best, and the last is the worst (Rajamani et al., 2021).

Before analysis and verification, according to the normative principle of index selection, the following index systems are set up (Thi et al., 2021).

Indicator 1: Grain self-sufficiency rate.


(3) $$S=\frac{T}{Q}\cdot 100\%$$

In Eq. (3), *S* represents the self-sufficiency rate of grain, *T* represents the total grain output of the place in that year, and *Q* represents the grain demand of the place in that year.

Indicator 2: *Per capita* possession of food.


(4) $$D=\frac{T}{C}$$

In Eq. (4), *D* represents the *per capita* possession of grain, *T* represents the total grain output of the place in that year, and *C* represents the permanent population of the place.

Indicator 3: The volatility of the total grain production in the area.


(5) $$P=\frac{T-F}{F}\cdot 100\%$$

In Eq. (5), *P* represents the volatility rate of the local total grain output, *T* represents the total grain output of the place in that year, and *F* represents the average grain output of the past 5 years.

According to Eqs. (3)–(5), the relevant food security evaluation index system can be clarified. The results are shown in Table 1.

**Table 1:** Food security evaluation index system.

Index system	Unit	Content	Index nature
Grain self-sufficiency rate	%	The ratio between total grain production and total grain consumption	Positive index
*Per capita* food	kg/per	Index to measure the fluctuation range of total grain output	Positive index
Volatility of total food production	%	Fluctuation of total grain output relative to the average output	Negative index

The specific calculation method of TOPSIS analysis method is as follows:

1. Determine the proportion of each evaluation index in each evaluation year.
(6) $$A_{ij}=\frac{x_{ij}}{\sum_{i=1}^{y}x_{ij}}$$
  In Eq. (6), *A_*ij*_* represents the raw data value of the *j*-th index in year *i*. *x_*ij*_* represents the dimensionless value of the *j*-th indicator in year *i*. *y* indicates the year.
2. Calculate the information entropy *H_*j*_* of the *j*-th evaluation index.
(7) $$B_{j}=-K\overset{y}{\underset{i=1}{\sum }}A_{ij}{\mathrm{lnA}}_{ij}$$
  In Eq. (7), *B_*j*_* represents the evaluation index information, *ln* is the natural logarithm, and the value of *K* is the reciprocal of the natural logarithmic year.
3. Calculate the difference coefficient *C_*j*_* of the *j* evaluation index.
(8) $$C_{j}=1-B_{j}$$
  In Eq. (8), *C_*j*_* represents the coefficient of variance of the evaluation index. *B_*j*_* represents the evaluation index information.
4. Calculate the index weight *E_*j*_*.
(9) $$E_{j}=\frac{C_{j}}{\sum_{j=1}^{n}C_{j}}$$
  In Eq. (9), *E_*j*_* represents the weight of the indicator, and *j* represents the *j*-th indicator.

## Analysis of the Results of the IoT Technology and TOPSIS Analysis Method on Food Security Innovation and Entrepreneurship

### Development Status of Food Security

#### Cultivated Land Area Over the Years

**Figure 4:**
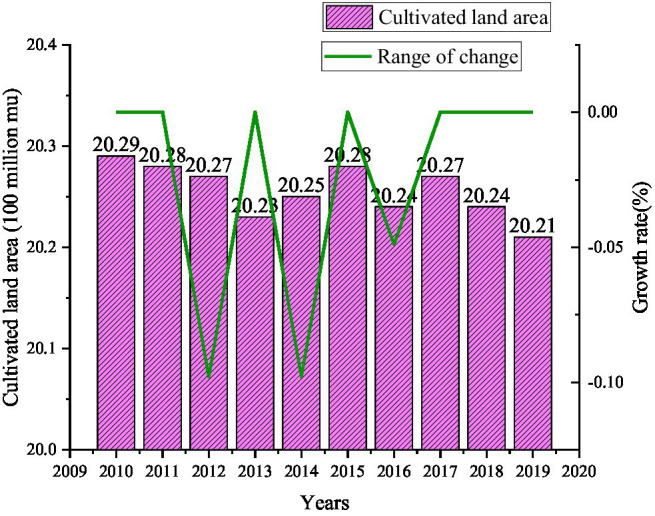
The area of arable land from 2010 to 2019.

Food is the material basis for human survival. In order to understand the current state of food security, the area of arable land is studied for food security, and the results are shown in Figure 4.

Figure 4 shows that from 2010 to 2019, the overall cultivated land area has shown a trend of shrinking. From 2010 to 2019, the area of arable land decreased by 0.33 million hectares from 135.27million hectares. From 2016 to 2019, it has been maintained at 134.93 million hectares of arable land.

#### Changes in Food Imports

**Figure 5:**
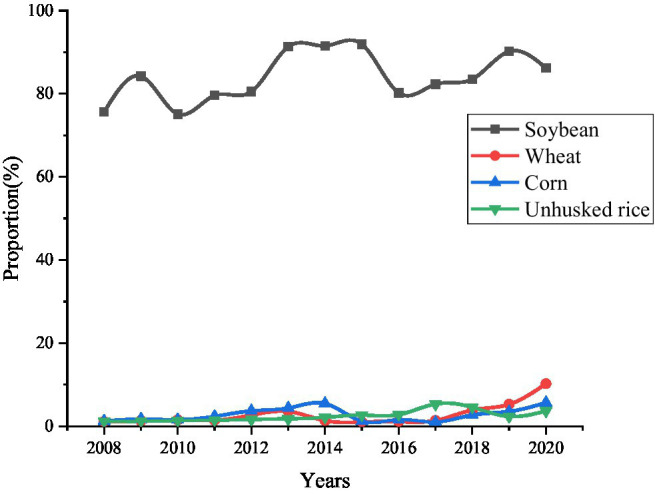
Import dependence on major agricultural products during 2008–2020.

According to the results of the seventh national census in May 2021, China has a population of approximately 1.412 billion. To solve the food problem of such a large population, it has to import from abroad. The amount of imported food is shown in Figure 5.

Figure 5 shows that from 2008 to 2020, among the soybean, corn, wheat, and rice crops, the import dependence of soybean has been at a high level, and the import volume of corn, wheat, and rice is not greatly different. According to relevant research reports, China’s annual demand for soybeans is about 110 million tons, but the domestic demand cannot meet such a large shortfall. About 90% of soybeans need to be imported from abroad. Among the imported soybeans, about 79% of soybeans will be processed into feed for livestock to eat, and about 21% of soybeans will be processed into edible oil to meet the edible needs of the masses. According to the prediction of relevant experts, the demand for grain in 2035 will be between 120 million and 210 million tons, so the issue of food security is imminent.

#### Total Domestic Grain Output Over the Years

**Figure 6:**
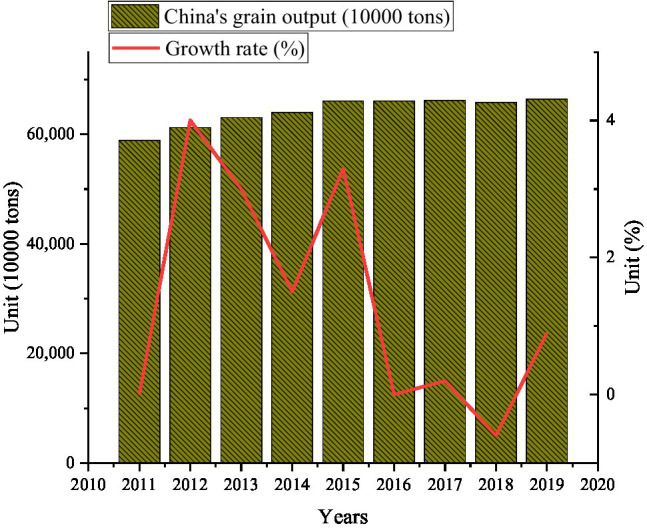
Grain production statistics and growth from 2011 to 2019.

While importing grain, the country has also continued to support and encourage the development of domestic agriculture. The domestic grain output from 2011 to 2019 is shown in Figure 6.

In Figure 6, the overall grain output from 2011 to 2019 showed a growing trend. Among them, the growth rate is the fastest from 2014 to 2015, about 3.3%. But grain production declined from 2017 to 2018, by about 3.715 million tons. Although the cultivated land area is continuously decreasing, the grain output is increasing compared with the cultivated land area from 2010 to 2019 in Figure 4. This data reflects the use of advanced technology in grain planting, or a certain improvement in planting methods.

#### The Status Quo of College Teachers and Students’ Perception of Food Security

**Figure 7:**
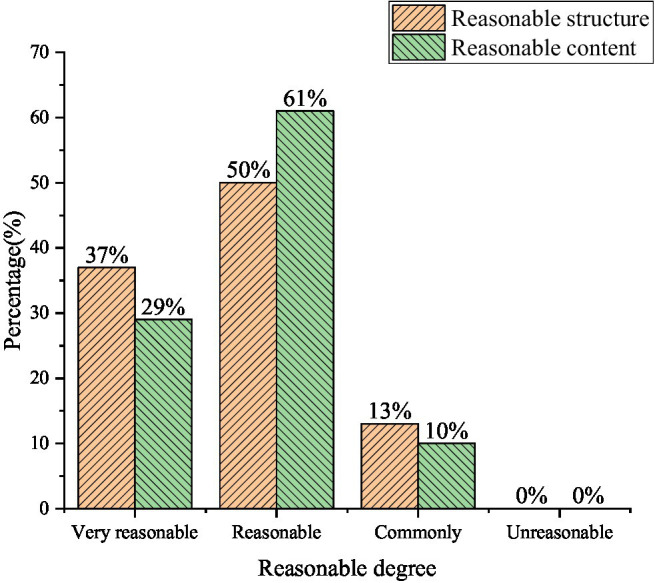
The frequency table selected in the validity test.

First, the validity test of the questionnaire was carried out. In order to ensure the validity of the questionnaire, whether the ideas and structure of the questions set in the *Questionnaire on Food Security Perceptions of University Teachers and Students* were reasonable, were submitted to 10 experts for validity testing. Ten experts retired, including six professors, three lecturers, and one teacher. They all evaluated the design and structure of the questionnaire, as shown in Figure 7.

In Figure 7, 87% of the experts believe that the questionnaire structure is very reasonable, and about 90% of the experts believe that the content of the questionnaire is reasonable. This shows that the questionnaire works well. Then, the questionnaire is tested for reliability. Because the span of the questionnaire is relatively long, it is not suitable to use the test–retest method for reliability testing. Therefore, the collected data is entered into Statistical Product and Service Solutions (SPSS) software. The responses to the questionnaire are tested for internal consistency (Cronbach), and the calculated result is 0.86. This indicates that the reliability of the questionnaire is high.

**Figure 8:**
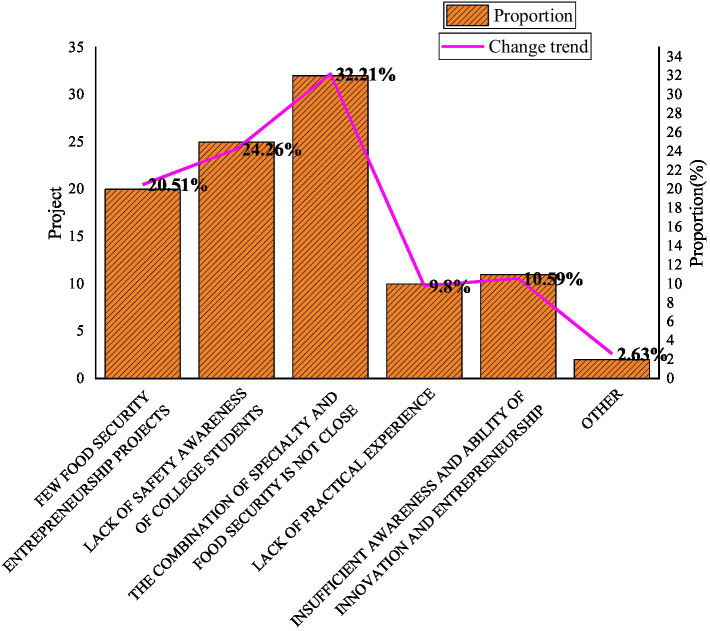
College teachers and students’ perception of food security.

College teachers and students are the main force in innovation and entrepreneurship research. A survey is conducted on the cognition of food security among college teachers and students, as shown in Figure 8.

Based on the arable land area, grain output and grain import volume over the years, it is obvious that with the rapid increase in population, food security has already faced serious challenges. It is everyone’s common expectation that high-quality teachers and students will join the development of food security. Figure 6 shows that 32.21% of teachers and students believe that the professional courses of finance and economics are not closely related to food security, and 24.26% of teachers and students do not have a deep understanding of food security. Around 20.51% of teachers and students think that the project of food security is lacking in innovation and entrepreneurship. Therefore, relevant management parties must pay close attention to these phenomena, plan the education programs of financial colleges and universities in time, introduce food security related topics into the classroom, and strengthen university teachers and students’ awareness of food security.

### An Empirical Analysis of TOPSIS Analysis Method for Food Security Innovation and Entrepreneurship Management

In order to help college teachers and students in their innovation and entrepreneurship in food security, the TOPSIS analysis method was used to calculate the index weights of relevant data. The data set from 2015 to 2020 was used as the sample of this food security indicator, and the 6-year safety management level was analyzed by combining these sample data with the TOPSIS analysis method. Finally, the 6-year data was sort, mainly to analyze the top five data. The results are shown in Table 2.

**Table 2:** Dimensionless processing results of evaluation index data.

Index	2015	2016	2017	2018	2019	2020
A	0.56	0.57	0.57	0.86	1	0.76
B	0.02	0.2	0.05	0.79	0.35	0.44
C	0.92	0.28	0.48	0	1	0.63
D	0.14	0	0.07	0.53	0.67	0.68
E	0.19	0.33	0.14	0	0.08	0.22

Next, the entropy method calculation should be performed on the dimensionless processing results of the evaluation index data according to the above equation, as shown in Table 3.

**Table 3:** Technique for Order Preference by Similarity to an Ideal Solution (TOPSIS) analysis method evaluation index weight calculation results.

Index	2015	2016	2017	2018	2019	2020
A	0.0406	0.02155	0.0236	0.0256	0.0536	0.0654
B	0.0411	0.2365	0.0463	0.3569	0.0694	0.2642
C	0.0549	0.0698	0.0412	0.0321	0.2635	0.2315
D	0.0521	0.03654	0.0569	0.3674	0.3066	0.0236
E	0.0365	0.0698	0.0352	0.0625	0.0325	0.0562

**Figure 9:**
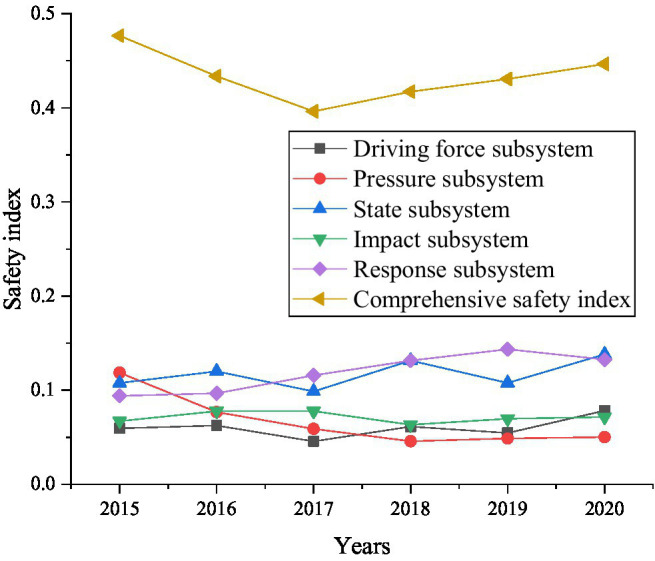
Changes in the food security index from 2015 to 2020.

According to Tables 2, 3, as well as the equation introduced above, the food security index from 2015 to 2020 can be obtained, as shown in Figure 9.

Food security are affected by many factors. In Figure 9, the overall food ecological security index showed a trend of first decline and then rise from 2015 to 2010. Among the driving force subsystem, pressure subsystem, state subsystem, influence subsystem, and response subsystem, only the safety index of the pressure subsystem has been declining. This shows that among the remaining five subsystems, the pressure subsystem does not make a significant contribution to the food security index.

**Figure 10:**
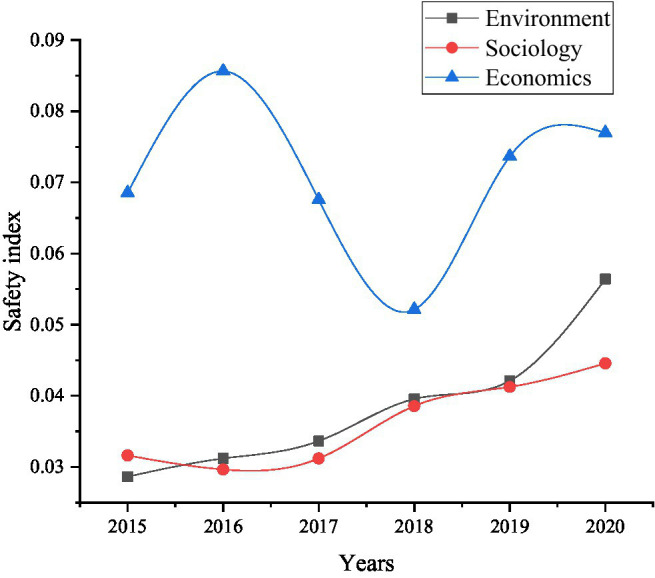
From 2015 to 2020, the change trend of the food security impact response subsystem index.

Figure 10 shows that in 2020 compared to 2015, the safety index of the response subsystem has increased by about 0.0389. In order to understand the specific reasons more clearly, experiment will be analyzed from three aspects: social factors, economic factors and environmental factors. The result is shown in Figure 10.

**Figure 11:**
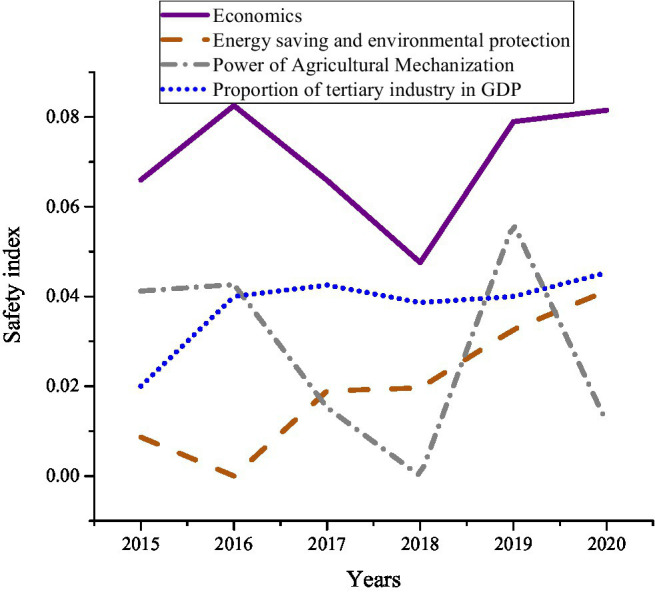
The change trend of the economic factor safety index of the food safety response subsystem during 2015–2020.

Figure 10 shows that among environmental factors, economic factors, and social factors safety indicators, economic factors fluctuate the most, indicating that the improvement of food security issues requires first raising the level of economic development. Meanwhile, environmental and social factors are constantly rising and changing. In order to have a clearer understanding of the reasons for the changes in economic factors, the experiment is compared the mechanical power of grain planting, energy conservation and environmental protection, the proportion of GDP and the economy. The results are shown in Figure 11.

In Figure 11, the factors of mechanical power, energy saving, and environmental protection, the proportion of GDP and the economy of grain planting are constantly changing. From 2016 to 2018, with the reduction of food planting machinery power, energy saving, and environmental protection investment and the impact of the tertiary industry, the safety indicators of economic factors showed a short-term decline. From 2018 to 2019, the continuous improvement in the level of planting machinery, the increase in energy conservation and environmental protection investment, and the rapid development of the tertiary industry stimulated the recovery of economic safety indicators. The investment ratio of mechanical power, energy saving, and environmental protection and the development level of the tertiary industry have a significant impact on the economic factors of the food security subsystem. Therefore, entrepreneurship in food security should not only pay attention to economic growth, but also consider the protection of the environment.

**Figure 12:**
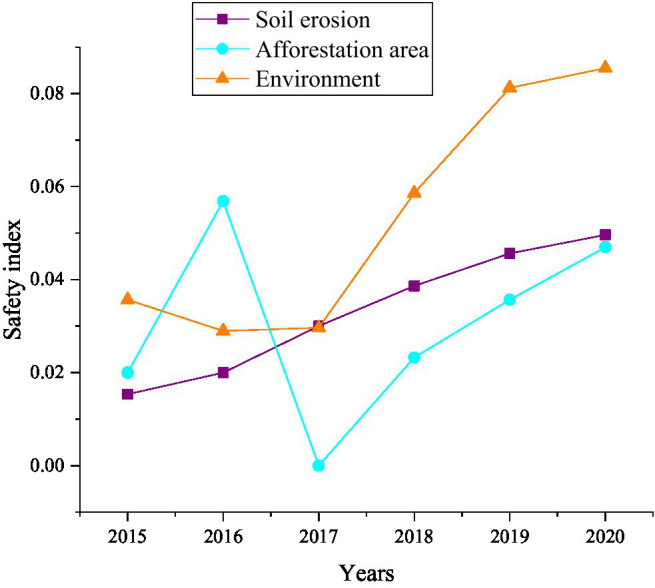
The change trend of the environmental factor safety index of food security response sub-systems from 2015 to 2020.

In order to clearly understand the reasons for the changes in environmental factors, comparisons were made from the three aspects of afforestation area, soil erosion area and environment. The results are shown in Figure 12.

Figure 12 shows that the environmental factor safety index of the food security response sub-system from 2015 to 2020 fluctuates and rises, and it is closely related to soil erosion and afforestation area. There is a positive correlation between them, that is, the increase of soil erosion will inevitably lead to the decline of environmental safety indicators, and the expansion of the afforestation area will certainly increase the environmental safety indicators, and vice versa.

In summary, Figures 9–12 show that economic factors have the greatest impact on food security. “The economic base determines the superstructure,” which tells the teachers and students of colleges and universities that when they choose to start businesses in food security, they should take economic factors as the focus of their work. Economic factors have been improved, then its impact on food security will be reduced. Meanwhile, protecting the natural environment is also the focus of innovation and entrepreneurship. The destruction of the ecological environment will definitely affect food security.

### IoT Technology in Food Security Innovation and Entrepreneurship

#### Application of IoT Technology in Food Growth

**Figure 13:**
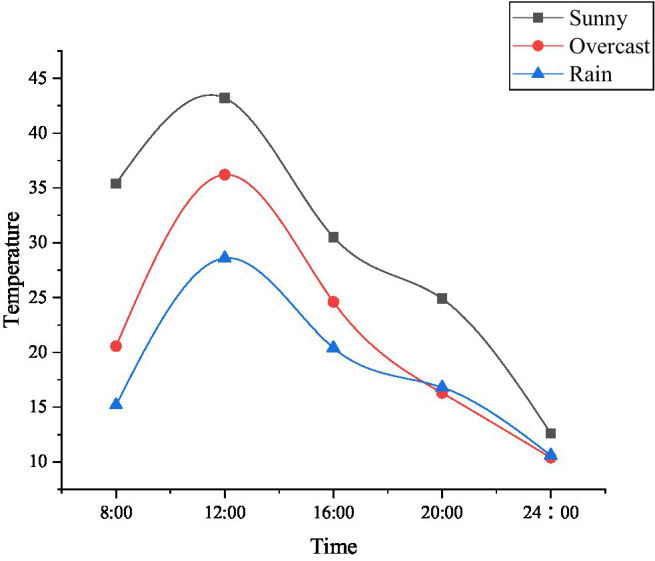
Temperature changes monitored by the Internet of Things (IoT) in greenhouse corn under different weather.

**Figure 14:**
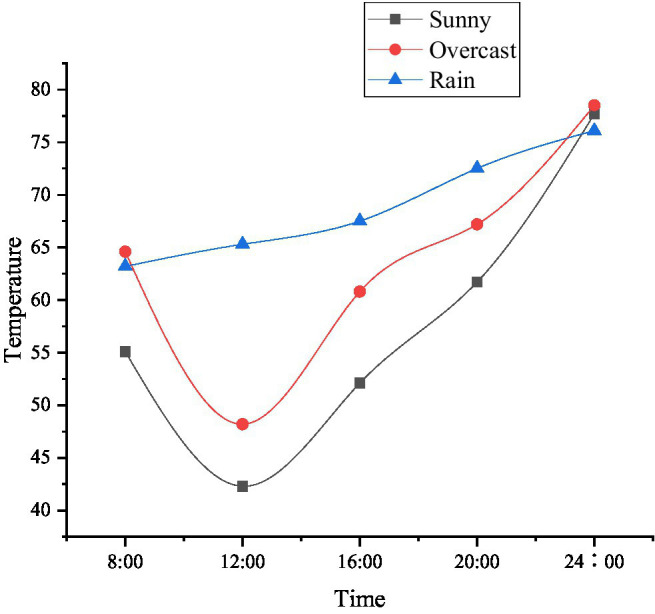
Humidity changes monitored by the IoT in greenhouse corn under different weather.

**Figure 15:**
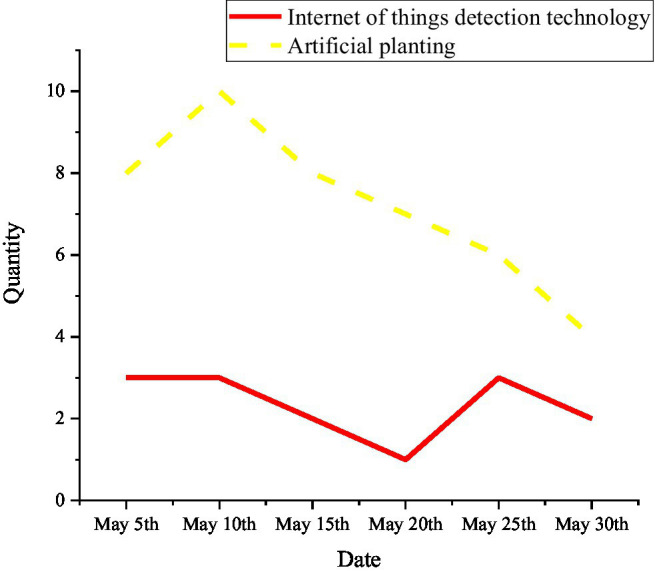
The death of plants and seedlings under artificial planting and IoT monitoring technology.

With the continuous improvement of science and technology, the application of the IoT technology in the food security has also been rapidly developed. The proposed method starts with the growth conditions of corn and analyzes the application of the IoT monitoring technology in the field of grain production, as shown in Figures 13–15.

The crops are inseparable from suitable temperature, humidity, and other external conditions during the growth process. The IoT monitoring technology can adjust various environments in the greenhouse according to different weather and time, to adapt to the entire growth process of corn. For example, the current temperature in the greenhouse is a bit hot for corn. The IoT monitoring technology uses Radio Frequency Identification (RFID) technology to obtain this signal, and it will immediately feed back to the monitoring personnel, which greatly reduces the death probability of the plant. Figure 13 indicates that the plant mortality rate monitored by the IoT is lower than that of workers relying on their own planting experience, and the plant mortality rate is reduced to 3.59%. Therefore, the monitoring technology of the IoT can be used in the innovation and entrepreneurship of university teachers and students in food security.

#### IoT Technology in Food Sales

**Figure 16:**
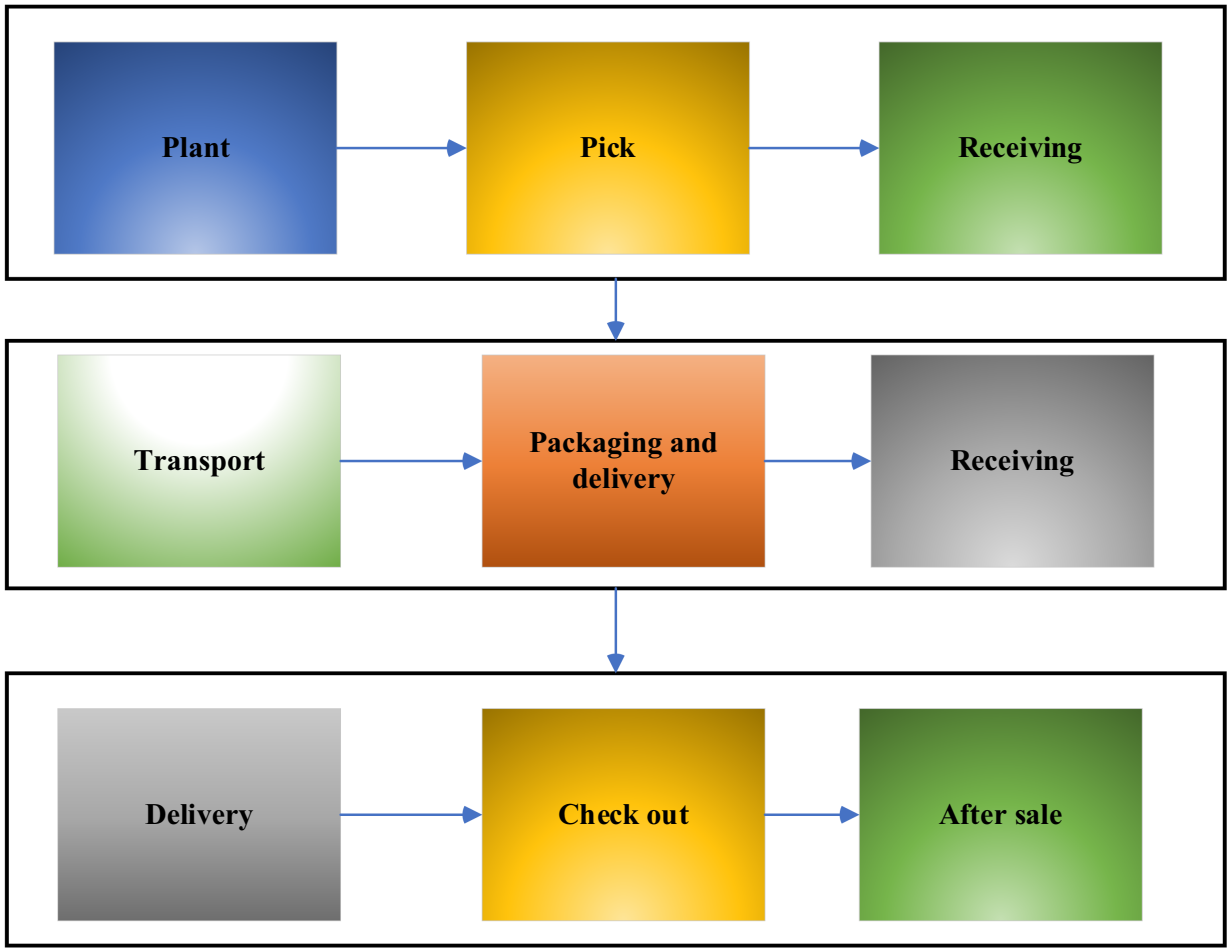
The process of food production from “production end to consumption end.”

**Figure 17:**
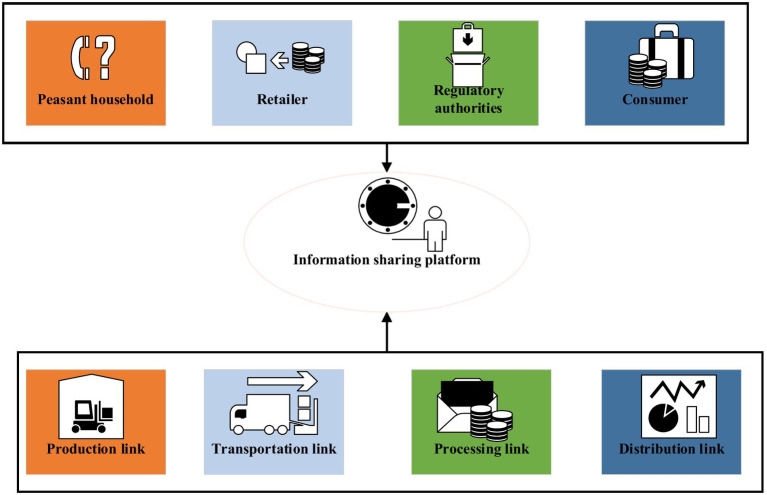
Internet of Things technology in the field of food sales.

The grain sales link can be roughly divided into three links: picking, sales, and after-sales. Each link can be divided into three smaller links, as shown in Figure 16. The application of IoT technology in grain sales is shown in Figure 17.

Figure 17 shows the entire process of food production, but the process of food production is complex and diverse. If the traditional offline sales are used, crop information asymmetry and grain accumulation may occur. The proposed method uses the monitoring technology of the IoT (Figure 17) to establish its own quality traceability system for each crop. Farmers can timely and accurately summarize all the information of the crop from germination, flowering, and finally to the consumer’s hands on the QR code of the crop. Consumers can scan the QR code with their mobile phone to view all the data of the food they bought. The detection technology of the IoT can not only realize information sharing, but also make consumers familiar with the food production process. Meanwhile, under the operation of the IoT technology, the healthy operation of the grain transportation, quality inspection, and after-sales links can be ensured, and the “one line” between suppliers and consumers can be achieved, and the common goal of reducing the cost of food sales can be achieved.

## Conclusion

With the continuous growth of the population, the rapid loss of rural labor force, and the frequent occurrence of natural disasters, food security has been constantly escalating and aggravating. In this study, food security was analyzed through the introduction of questionnaires, TOPSIS analysis, and IoT monitoring technology.

These conclusions were drawn: at present, teachers and students in colleges and universities lack awareness of food security. TOPSIS analysis concluded that the core of solving food security problems lies in economic factors. Teachers and students in colleges and universities should pay attention to the changes in economic factors when starting businesses in food security. The application of IoT monitoring technology in food growth and sales can greatly promote the growth of crops and the long-term development of food security. Crop growth can reduce seedling mortality. Food sales can guarantee its quality and so on. This provides a new strategy for subsequent entrepreneurship and innovation. Due to limited energy, the economics of food security have not been specifically discussed. In the follow-up, the economic benefits of the plan will be evaluated according to the specific situation, so that the plan can better promote the long-term development of food security.

## Data Availability Statement

The raw data supporting the conclusions of this article will be made available by the authors, without undue reservation.

## Ethics Statement

The studies involving human participants were reviewed and approved by Henan University of Technology Ethics Committee. The patients/participants provided their written informed consent to participate in this study. Written informed consent was obtained from the individual(s) for the publication of any potentially identifiable images or data included in this article.

## Author Contributions

The author confirms being the sole contributor of this work and has approved it for publication.

## Funding

This work was supported by the National Social Science Foundation of China (20BJY152), the Innovation Funds Plan of Henan University of Technology (2021-SKCXTD-17), and the Cultivation Programme for Young Backbone Teachers in Henan University of Technology.

## Conflict of Interest

The author declares that the research was conducted in the absence of any commercial or financial relationships that could be construed as a potential conflict of interest.

## Publisher’s Note

All claims expressed in this article are solely those of the authors and do not necessarily represent those of their affiliated organizations, or those of the publisher, the editors and the reviewers. Any product that may be evaluated in this article, or claim that may be made by its manufacturer, is not guaranteed or endorsed by the publisher.
